# Linear Parameter Varying Identification of Dynamic Joint Stiffness during Time-Varying Voluntary Contractions

**DOI:** 10.3389/fncom.2017.00035

**Published:** 2017-05-19

**Authors:** Mahsa A. Golkar, Ehsan Sobhani Tehrani, Robert E. Kearney

**Affiliations:** Department of Biomedical Engineering, McGill UniversityMontréal, QC, Canada

**Keywords:** joint stiffness, ankle biomechanics, system identification, time-varying, linear parameter varying

## Abstract

Dynamic joint stiffness is a dynamic, nonlinear relationship between the position of a joint and the torque acting about it, which can be used to describe the biomechanics of the joint and associated limb(s). This paper models and quantifies changes in ankle dynamic stiffness and its individual elements, intrinsic and reflex stiffness, in healthy human subjects during isometric, time-varying (TV) contractions of the ankle plantarflexor muscles. A subspace, linear parameter varying, parallel-cascade (LPV-PC) algorithm was used to identify the model from measured input position perturbations and output torque data using voluntary torque as the LPV scheduling variable (SV). Monte-Carlo simulations demonstrated that the algorithm is accurate, precise, and robust to colored measurement noise. The algorithm was then used to examine stiffness changes associated with TV isometric contractions. The SV was estimated from the Soleus EMG using a Hammerstein model of EMG-torque dynamics identified from *unperturbed* trials. The LPV-PC algorithm identified (i) a non-parametric LPV impulse response function (LPV IRF) for intrinsic stiffness and (ii) a LPV-Hammerstein model for reflex stiffness consisting of a LPV static nonlinearity followed by a time-invariant state-space model of reflex dynamics. The results demonstrated that: (a) intrinsic stiffness, in particular ankle elasticity, increased significantly and monotonically with activation level; (b) the gain of the reflex pathway increased from rest to around 10–20% of subject's MVC and then declined; and (c) the reflex dynamics were second order. These findings suggest that in healthy human ankle, reflex stiffness contributes most at low muscle contraction levels, whereas, intrinsic contributions monotonically increase with activation level.

## 1. Introduction

Ankle joint biomechanics can be described by the relationship between the joint position and the torque acting about it, defined as *dynamic joint stiffness*. It describes the properties of the human actuator and determines (a) the internal load that the central nervous system (CNS) must control and (b) the joint behavior in response to external loads or perturbations. Consequently, a quantitative knowledge of joint stiffness is essential for understanding the normal control of posture and movement and the nature of motor function disorders such as spasticity, rigidity, hypertonia, hypotonia, and flaccidity (Amato and Ponziani, 1999; Bar-On et al., 2014). Also, a good model of joint stiffness is invaluable for the design and control of ankle prostheses and orthoses (Palazzolo et al., 2007).

Joint stiffness modeling has been extensively investigated in the literature (e.g., Kearney et al., 1997; Mirbagheri et al., 2000; Jalaleddini and Kearney, 2011; Sobhani Tehrani et al., 2014). Two distinct physiological mechanisms contribute to joint stiffness: (i) Limb inertia, viscoelasticity of muscle-tendon complex, and active properties of muscle contraction that together define *intrinsic* stiffness; and (ii) Stretch reflex feedback that changes muscle activation in response to changes in muscle length leading to *reflex* stiffness. At the human ankle, this has been efficiently modeled with a Parallel-Cascade (PC) structure having separate pathways for intrinsic and reflex stiffness (Kearney et al., 1997). This study showed that under quasi-stationary conditions, where the joint is perturbed around an operating point (OP) defined by joint position and activation level, the intrinsic stiffness can be modeled by an impulse response function (IRF) and the nonlinear reflex stiffness can be modeled by a Hammerstein system consisting of a static nonlinearity followed by a linear dynamics.

However, numerous quasi-stationary studies, using system identification techniques, demonstrated that both intrinsic and reflex stiffness parameters change drastically and systematically with ankle position and activation level (Weiss et al., 1986; Sinkjaer et al., 1988; Carter et al., 1990; Mirbagheri et al., 2000; Van der Helm et al., 2002; Bar-On et al., 2014; Jalaleddini et al., 2016). Thus, in many functional tasks, like normal gait, where joint position and neural activation continuously change to control movement and counteract external perturbations, joint stiffness will exhibit time-varying (TV) behavior. Furthermore, there is evidence that this TV behavior cannot be predicted simply by interpolating local TI models identified under quasi-stationary conditions (Kirsch and Kearney, 1997). Therefore, more advanced methodologies are required to identify and characterize joint stiffness during movement or functional tasks.

To this end, a number of approaches have been proposed and used over the years. These include intramuscular mechanism modeling using optimization that minimizes a predefined cost function (Sartori et al., 2015), system identification techniques, or a combination of both (de Vlugt et al., 2010). Methods for identification of TV systems can be divided into four main categories: (i) short segment, (ii) ensemble-based, (iii) time-varying, and (iv) linear parameter varying (LPV).

Short segment methods (Ludvig and Perreault, 2012; Rouse et al., 2014; Jalaleddini et al., 2017) divide non-stationary data into a number of segments with quasi-stationary behavior and identify a time-invariant model for each segment. The segmentation is not always trivial and often requires the TV behavior to be very slow. Ensemble-based methods (MacNeil et al., 1992; Kirsch et al., 1993; Ludvig et al., 2011; Lee and Hogan, 2015) are effective but require many trials with *identical* TV behavior, which is hard to achieve in many experimental conditions. Moreover, repeating the same task many times may result in fatigue and affect the reliability of estimates. Time-varying identification techniques (Sanyal et al., 2005; Ikharia and Westwick, 2006, 2007; Guarin and Kearney, 2015) use temporal expansion to estimate how the system parameters change continuously with time using data from a single trial; thus simplifying data requirements significantly. However, selecting proper basis functions for temporal expansion is often difficult and the number of model parameters increases significantly if the time-dependent changes are fast; thus reducing the quality of the estimates. Moreover, none of the models identified by these methods can predict the system response to novel trajectories.

LPV models have a structure resembling that of linear systems whose parameters change as functions of one or more time-dependent signal called scheduling variables (SV). As such, the LPV structure is an excellent candidate for modeling joint stiffness during functional tasks where the TV behavior is mostly due to dependency on neuromuscular variables that vary with time. Also, by relating TV behavior to SVs rather than time, LPV models model the nonlinear mechanisms that generate the TV behavior and thus have the ability to predict the response to novel trajectories. Finally, control theory is well developed for LPV systems (Mohammadpour and Scherer, 2012), which makes LPV models suitable for prostheses and orthoses control.

Despite the significant advantages of LPV models, methods for LPV identification of nonlinear physiological systems have not been studied much. Examples include the LPV modeling of glucose-insulin dynamics in type I diabetes (Cerone et al., 2012) and of the hemodynamic response to profiled hemodialysis (Javed et al., 2010). Our lab has pioneered the use of LPV methods for the identification of joint stiffness. Specifically, Sobhani Tehrani et al. (2013a) identified a LPV mass-spring-damper (LPV IBK) model of intrinsic ankle joint stiffness for imposed movements at rest. Soon after, Van Eesbeek et al. (2013) used a LPV subspace method to identify time-variant intrinsic impedance of the human wrist joint. Subsequently, Sobhani Tehrani et al. (2014) developed subspace LPV parallel-cascade (LPV-PC) method for the identification of both intrinsic and reflex stiffness during large passive ankle movements. However, these studies were conducted under passive (i.e., at rest) conditions and quantified position dependent changes in stiffness. The study of joint stiffness changes during large time-varying muscle contractions is challenging since neither the muscle activation level nor the voluntary torque are directly measurable as scheduling variable.

In this work, we used the subspace LPV-PC algorithm (Sobhani Tehrani et al., 2014) to characterize changes in both intrinsic and reflex stiffness during isometric, time-varying contractions of the ankle plantarflexors of healthy human subjects. This algorithm, models the intrinsic pathway as a non-parametric LPV impulse response function (LPV IRF) and reflex stiffness as a LPV-Hammerstein cascade of a LPV static nonlinearity and a time invariant (TIV) linear dynamics. The reflex linear dynamic was assumed TIV, similar to previous works (Sinkjaer et al., 1996, 1988; Ludvig et al., 2011). The scheduling variable, the joint voluntary torque, was estimated from EMG signals using a time-invariant Hammerstein model of EMG-Torque dynamics, which was previously identified using an error-in-variable subspace algorithm. In addition to the experimental examination of the subspace LPV-PC identification method, we also performed Monte-Carlo simulations to demonstrate its accuracy and precision.

## 2. Methods

### 2.1. Problem formulation

**Figure 1:**
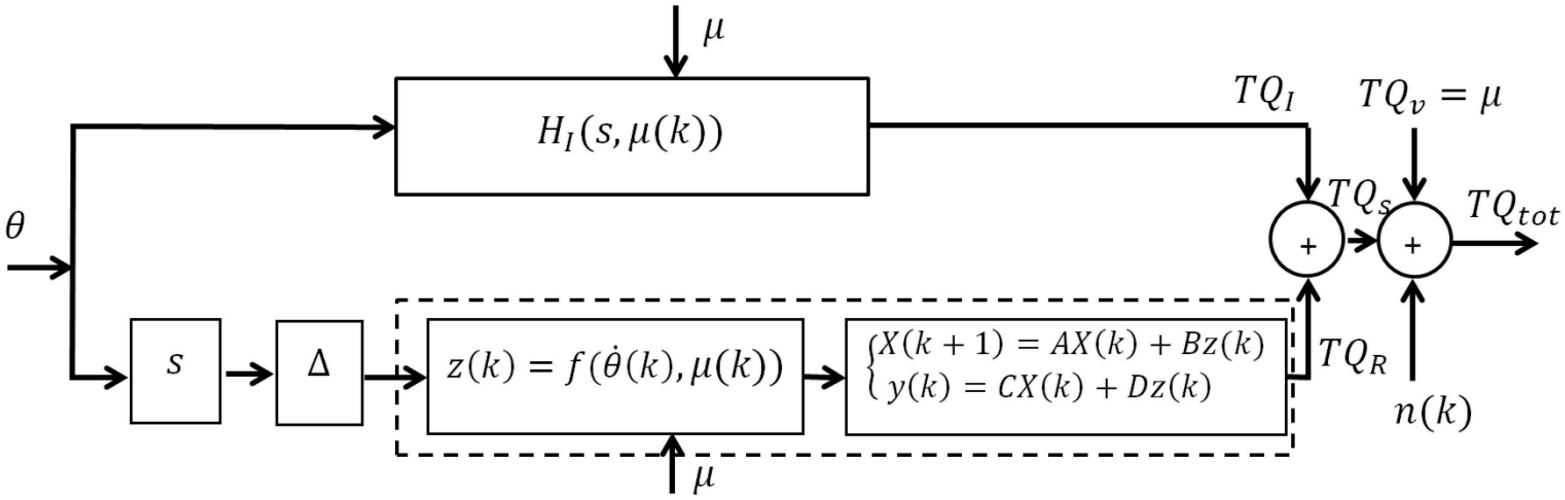
**Subspace LPV Parallel-Cascade (LPV-PC) model of joint dynamic stiffness**.

Figure 1 shows a block diagram of the subspace LPV-PC model with joint angle as input (θ), total torque as output (*TQ*_*tot*_), and voluntary torque as scheduling variable (μ). The total torque is the sum of intrinsic (*TQ*_*I*_), reflex (*TQ*_*R*_), and voluntary torques (*TQ*_*V*_), and the colored measurement noise (*n*). This can be written as:

(1) $$TQ_{tot}(k)=TQ_{I}(k)+TQ_{R}(k)+TQ_{V}(k)+n(k)$$

and the stiffness torque is:

(2) $$TQ_{s}(k)=TQ_{I}(k)+TQ_{R}(k)$$

where,

(3) $$\begin{array}{l} TQ_{s}={\left[TQ_{s}(0)\ldots TQ_{s}(N-1)\right]}^{T}\\ TQ_{I}={\left[TQ_{I}(0)\ldots TQ_{I}(N-1)\right]}^{T}\\ TQ_{R}={\left[TQ_{R}(0)\ldots TQ_{R}(N-1)\right]}^{T} \end{array}$$

(4) $$E={\left[n(0)\ldots n(N-1)\right]}^{T}$$

and *N* represents the total number of samples. The intrinsic stiffness is represented by a LPV IRF model:

(5) $$TQ_{I}(k)=\sum_{l=-L}^{l=L}h_{l}(\mu (k))\theta (k-l)$$

where *h*_*l*_ are the IRF weights that are functions of SV (μ(*k*)) represented by a basis expansion on the SV:

(6) $$h_{l}≜\sum_{j=0}^{n_{i}}h_{lj}g_{j}(\mu (k))$$

where *h*_*ij*_ is the (*i,j*)-th coefficient for the *i*-th lag of IRF, *g*_*j*_ represents the *j*-th basis expansion of the SV and *n*_*i*_ is the expansion order. Now, rewrite this equation in matrix form to obtain a data equation for the intrinsic pathway; the unknown intrinsic stiffness parameters are:

(7) $$\beta_{I}={\left[H_{-L}\ldots H_{l}\ldots H_{+L}\right]}^{T}$$

where *H*_*l*_ contains the LPV IRF weights for lag *l*,

(8) $$H_{l}={\left[h_{l_{0}}\ldots h_{ln_{i}}\right]}^{T}$$

The basis expansion of the SV can be represented in vector form:

(9) $$G_{i}(k)={\left[g_{0}(\mu (k))\ldots g_{n_{i}}(\mu (k))\right]}^{T}$$

and the lagged position inputs with the vector:

(10) $$\Theta (k)={\left[\theta (k+l)\ldots \theta (k)\ldots \theta (k-l)\right]}^{T}$$

Then, the input to the intrinsic pathway is constructed by the Kronecker product of Equations (9, 10):

(11) $$U_{I}(k)=\Theta (k)⊗G_{i}(k)$$

Now, rewriting Equation (5) in vector form, the data equation for the intrinsic pathway is:

(12) $$TQ_{I}=\Psi_{I}\beta_{I}$$

with the regressor:

(13) $$\Psi_{I}={\left[U_{I}(L)\ldots U_{I}(N-1-L)\right]}^{T}$$

The reflex stiffness is modeled by a differentiator, a delay, and a Hammerstein system comprising a LPV static nonlinearity followed by a time-invariant linear state-space model. The input to the Hammerstein system is the delayed joint velocity (due to reflex delay) denoted by *dvel* in the equations. The output of the static nonlinearity is approximated by an orthonormal basis function expansion of the Hammerstein system input, *dvel*:

(14) $$\begin{array}{l} z(k)=f(dvel(k),\mu (k))≃\sum_{i=0}^{n_{p}}\omega_{i}(\mu (k))g_{i}(dvel(k))\\ \text{ where},\\ \;\omega_{i}=\sum_{j=0}^{n_{r}}\omega_{ij}g_{j}(\mu (k)) \end{array}$$

and *g*_*i*_(*dvel*(*k*)) is the *i*-th basis expansion of reflex input (*dvel*), *g*_*j*_(μ(*k*)) is the *j*-th basis expansion of the SV, and ω_*ij*_ is the coefficient of their products; *n*_*p*_ and *n*_*r*_ are the expansion orders of the input (*dvel*) and the SV, respectively. Thus, using basis expansions of the input, the static nonlinearity is converted to *n*_*p*_ parallel linear functions, where the expansion weights are dependent on the SV. The vectors of input and SV basis expansions, for reflex pathway, can be written as:

(15) $$\begin{array}{l} \;G_{r}(k)={\left[g_{0}(\mu (k))\ldots g_{n_{r}}(\mu (k))\right]}^{T}\\ DV(k)={\left[g_{0}(dvel(k))\ldots g_{n_{p}}(dvel(k))\right]}^{T} \end{array}$$

with unknown parameters:

(16) $$\begin{array}{l} \;\Omega ={\left[\Omega_{0}\ldots \Omega_{n_{p}}\right]}^{T}\\ \Omega_{i}={\left[\omega_{i0}\ldots \omega_{in_{r}}\right]}^{T} \end{array}$$

Thus, the input to reflex linear dynamics becomes:

(17) $$U_{R}(k)=DV(k)⊗G_{r}(k)$$

The linear system is modeled using a discrete-time state-space representation of order *m*:

(18) $$\begin{array}{l} X(k+1)=AX(k)+Bz(k)\\ \;TQ_{R}(k)=CX(k)+Dz(k) \end{array}$$

where *X*(*k*) is the state vector, *z*(*k*) is the input to reflex linear dynamics, and *A*, *B*, *C*, and *D* are the state-space matrices and:

(19) $$B={\left[b_{1}\ldots b_{m}\right]}^{T},\;\;\;\;\;\;D=[d]$$

Substituting Equation (17) in Equation (18) yields:

(20) $$\begin{array}{l} X(k+1)=A_{R}X(k)+B_{\Omega }U_{R}(k)\\ \;TQ_{R}(k)=C_{R}X(k)+D_{\Omega }U_{R}(k) \end{array}$$

where,

(21) $$\begin{array}{l} B_{\Omega }=B⊗\Omega =\left[\begin{array}{ccc} b_{1}\Omega_{0}^{T} & \ldots & b_{1}\Omega_{n_{p}}^{T}\\ \vdots & \ddots & \vdots \\ b_{m}\Omega_{0}^{T} & \ldots & b_{m}\Omega_{n_{p}}^{T} \end{array}\right],\\ D_{\Omega }=D⊗\Omega =\left[\begin{array}{c} d\Omega_{0}^{T}\ldots d\Omega_{n_{p}}^{T} \end{array}\right] \end{array}$$

Combining the data equations for intrinsic and reflex pathways (Equations 12, 20), the total joint stiffness can be represented with a *Multi-Input-Single-Output* (MISO) state-space model:

(22) $$\begin{array}{l} X(k+1)=A_{R}X(k)+B_{T}U_{T}(k)\\ \;{\hat{TQ}}_{s}(k)=C_{R}X(k)+D_{T}U_{T}(k)+n(k) \end{array}$$

where,

(23) $$U_{T}(k)=\left[\begin{array}{cc} U_{R}(k) & U_{I}(k) \end{array}\right]$$

(24) $$\begin{array}{l} \;B_{T}=\left[\begin{array}{cc} B_{\Omega } & \underset{(2L+1)n_{i}\text{columns}}{\underset{︸}{0\ldots 0}} \end{array}\right]\\ D_{T}=\left[\begin{array}{cc} D_{\Omega } & \beta_{I} \end{array}\right] \end{array}$$

### 2.2. Subspace LPV-PC identification algorithm

An orthogonal projection algorithm (Sobhani Tehrani et al., 2014; Jalaleddini et al., 2016) was used to first decompose intrinsic and reflex torque components and subsequently estimate the unknown model parameters. The unknown parameters to estimate are (i) the intrinsic IRF parameters (β_*I*_ in Equation 7); (ii) the reflex non-linearity coefficients (Ω in Equation 16); and (iii) the reflex linear system matrices *A*, *B*, *C*, and *D* in Equation (18). This can be achieved through the following steps:

1. Construct the input signal *U*_*T*_(*k*) from Equation (23).
2. Use the *Past Input-Multivariable Output Error State Space* algorithm (PI-MOESP) (Verhaegen and Dewilde, 1992) with input and output signals (*U*_*T*_(*k*) and *TQ*_*s*_(*k*)) to estimate the order of the system (Equation 22), *m*.
3. Construct the extended observability matrix using *m* and the input and output signals, and use it to estimate the state-space matrices Â_*R*_ and Ĉ_*R*_.
4. Form the data equation, and isolate the intrinsic and reflex parameters (β_*I*_, β_*R*_) in separate terms:
  (25) $$\hat{TQ_{s}}=TQ_{I}+TQ_{R}+E=\Psi_{I}\beta_{I}+\Psi_{R}\beta_{R}+E$$
  where, Ψ_*I*_ and β_*I*_ are defined in Equations (7, 13), respectively, and:
  $$\begin{array}{l} \Psi_{R}=\left[\begin{array}{cc} 0 & U_{R}^{T}(0)\\ \vdots & \vdots \\ \sum_{\tau =0}^{N-2}U_{R}^{T}(\tau )⊗{\hat{C}}_{R}{\hat{A}}_{R}^{N-2-\tau } & U_{R}^{T}(N-1) \end{array}\right]\\ \;\beta_{R}={\left[B^{T}d\right]}^{T}⊗\Omega \end{array}$$
5. Use orthogonal projection to decompose the total torque into its intrinsic and reflex components:
  $$\begin{array}{l} \;{\hat{TQ}}_{I}=(I-{\Psi_{I}}^{†}\Psi_{R}{\Psi_{R}}^{†}\Psi_{I}{)}^{†}{\Psi_{I}}^{†}(I-\Psi_{R}{\Psi_{R}}^{†}){\hat{TQ}}_{s}\\ {\hat{TQ}}_{R}={\hat{TQ}}_{s}-\Psi_{I}\hat{\beta_{I}} \end{array}$$
6. Use the subspace Hammerstein method described in Sobhani Tehrani et al. (2013b) to estimate the reflex pathway model using *dvel*(*k*) as input and ${\hat{TQ}}_{R}\left(k\right)$ as output.

## 3. Simulation study

### 3.1. Methods

We evaluated the performance of the subspace LPV-PC identification algorithm using a simulation study of the LPV-PC model of human's ankle stiffness dynamics (Figure 1). All parameter and nominal values of simulation model were selected based on experimental results reported in literature (Mirbagheri et al., 2000; Jalaleddini et al., 2016).

#### 3.1.1. Model

The intrinsic stiffness was simulated as the LPV IBK model:

(26) $$TQ_{I}(k)=I\ddot{\theta }(k)+B\dot{\theta }(k)+K(\mu (k))\theta (k)$$

**Figure 2:**
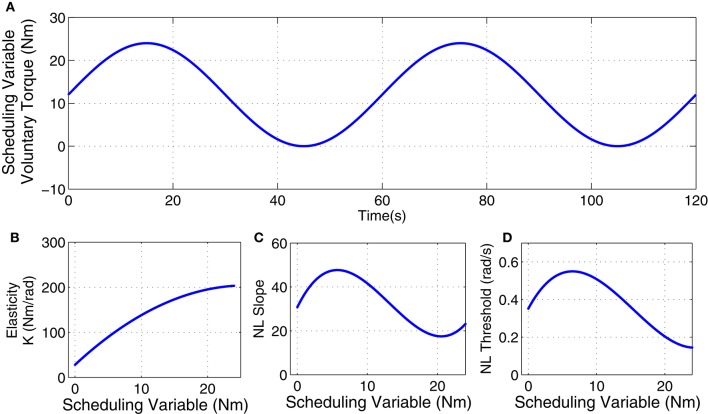
**Simulated parameters: (A)** voluntary torque (scheduling variable), **(B)** intrinsic Elasticity (*K*), **(C)** reflex nonlinearity gain, and **(D)** reflex nonlinearity threshold variation with scheduling variable.

The inertia (*I*) and viscosity (*B*) were set to 0.015 Nm.s^2^/rad and 1.1 Nm.s/rad. The intrinsic elastic parameter (*K*) and reflex gain and threshold were simulated to have a non-linear behavior with changes in voluntary torque (SV). The linear dynamics of reflex pathway was assumed TIV. Figure 2 demonstrates the simulated parameters. Elasticity was modeled as a polynomial of order 3 for SV. The reflex gain (represented as NL slope in Figure 2C) and threshold (NL threshold, Figure 2D) of reflex Hammerstein system were modeled as polynomial of order 6 for input and a polynomial of order 4 for the SV.

The linear dynamic element of the reflex pathway was assumed to be a second-order low-pass filter with the dynamics:

(27) $$H(s)=\frac{G\omega_{n}^{2}}{s^{2}+2s\zeta \omega_{n}+\omega_{n}^{2}}$$

where *G* = 1 is the system gain, ω_*n*_ = 25 rad/s is the natural frequency and ζ = 0.9 rad/s is the damping factor. The reflex delay was assumed to be 40 ms. This system was simulated using MATLAB Simulink at 1 kHz for 120 s.

#### 3.1.2. Input and noise

The input signal was a pseudo random arbitrary level distributed signal (PRALDS) with random switching time uniformly distributed over [250, 350] ms, and maximum amplitude equal to 0.05 rad. This input signal was then filtered with a second order Butterworth low-pass filter with cutoff frequency of 30 Hz to represent the actuator dynamics.

Output noise was modeled as a white Gaussian signal filtered with a second order Butterworth low-pass filter with cutoff frequency equal to 15 Hz. The noise amplitude was adjusted to produce an average signal-to-noise ratio (SNR) of 10 dB. SNR was calculated as:

(28) $$\text{SNR}(\text{dB})=20{\text{log}}_{10}\left(\frac{{\text{RMS}}_{\text{signal}}}{{\text{RMS}}_{\text{noise}}}\right)$$

**Figure 3:**
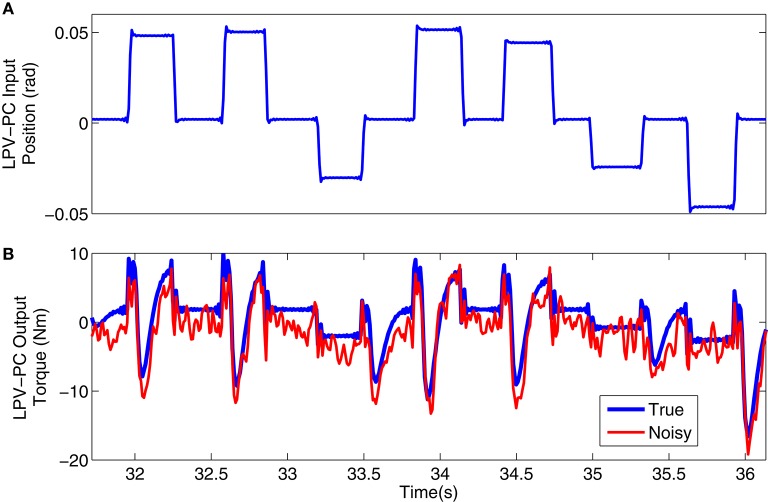
**Typical simulation signal used for LPV-PC identification: (A)** position input, **(B)** noise-free (blue) and noisy (red) torque output.

**Figure 4:**
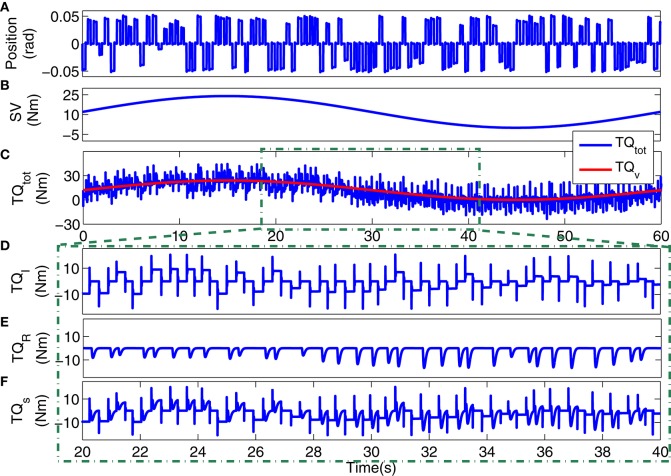
**Typical simulation data: (A)** position input, **(B)** voluntary torque (SV), **(C)** total torque (*TQ*_*tot*_) and SV (*TQ*_*v*_), 20s segments of: **(D)** intrinsic stiffness, **(E)** reflex stiffness, **(F)** stiffness torque.

Figure 3 shows a 4s segment of the position input and noise free and noisy output data, and Figure 4 shows the simulated input (position), scheduling variable (voluntary torque), and torques.

#### 3.1.3. Analysis

To avoid aliasing, all simulation data were filtered with an eighth-order low-pass filter with cutoff frequency of 45 Hz and decimated to 100 Hz before analysis. The intrinsic pathway was identified using a LPV IRF model as described by Equation (5). We calculated the equivalent elasticity of the identified model as the low-frequency (or DC) gain of the LPV IRFs at each SV snapshot. This gain is the steady state value of the integral of identified intrinsic LPV IRF at each SV snapshot.

We assessed the quality of fit by calculating the variance accounted for (VAF):

(29) $$\%VAF=\left[1-\frac{\sum_{i=1}^{N}{\left(TQ_{i}-{\hat{TQ}}_{i}\right)}^{2}}{\sum_{i=1}^{N}TQ_{i}^{2}}\right]\times 100$$

where *TQ*_*i*_ represents the noise free simulated torque at time interval *i* and $\hat{TQ}$ represented the estimated value; *N* is the number of samples.

We quantified the quality of identification estimates by using 200 Monte-Carlo trials, each having a new realization of input and noise. The bias and random errors for reflex static nonlinearity estimates were calculated as:

(30) $$\begin{array}{l} \text{ Bias Error}=\rho -E(\hat{\rho })\\ \text{Random Error}=E{\left(\hat{\rho }-E(\hat{\rho })\right)}^{2} \end{array}$$

where ρ and $\hat{\rho }$ represent true and estimated parameter respectively. Note that both Bias Error and Random Error are also functions of delayed velocity and SV.

### 3.2. Results

**Figure 5:**
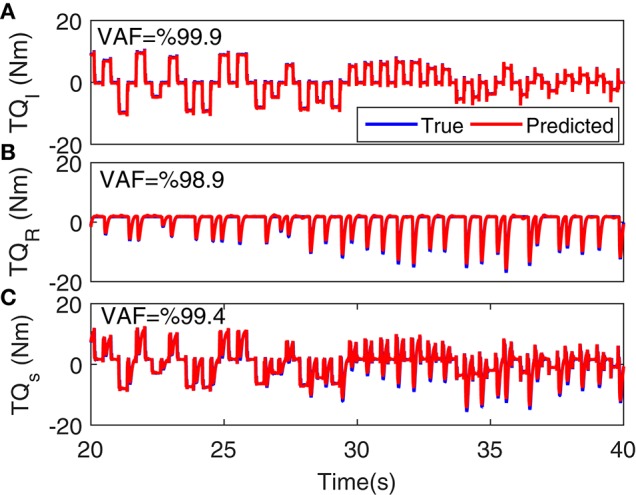
**Torque prediction for (A)** intrinsic stiffness, **(B)** reflex stiffness, and **(C)** total stiffness, for a typical trial. A 20s segment of data with largest variation in voluntary torque (i.e., SV) is presented for better visualization. VAFs confirmed the accuracy of method in identifying the simulated model.

**Figure 6:**
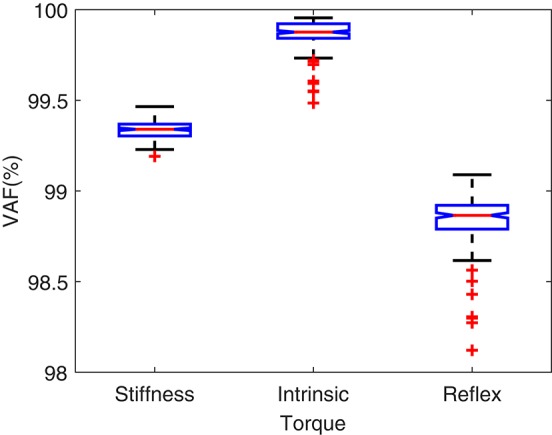
**VAF for torque predictions in 200 Monte-Carlo simulation trials**.

Figure 5 shows the torque prediction profiles for a typical trial. The subspace LPV-PC identification algorithm used, identified the simulated model very accurately as confirmed by high VAFs calculated for each pathway. Figure 6 summarizes the torque prediction accuracy for each pathway as well as the stiffness torque, for 200 Monte-Carlo trials identified, in boxplot representation. The VAFs were always above 98% for the high noise level tested in this simulation study, confirming the efficiency of method in decomposing the total torque into intrinsic and reflex contributions.

**Figure 7:**
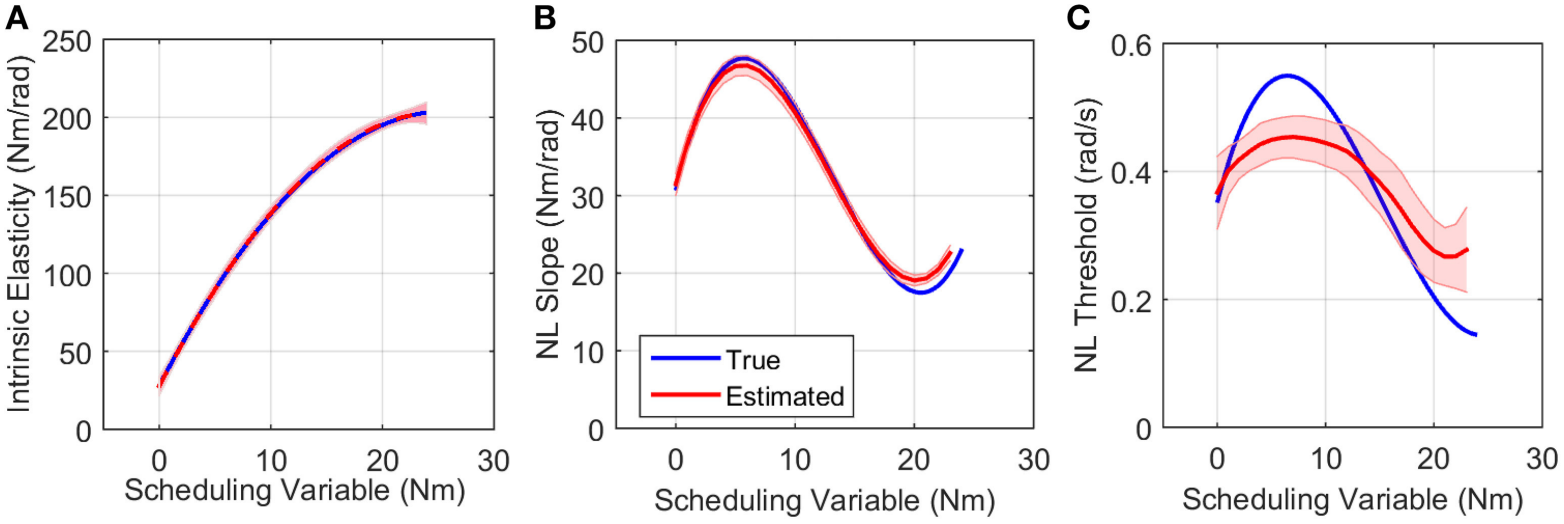
**True (blue) and the mean of estimated (red) (A)** intrinsic elasticity, **(B)** reflex LPV-static nonlinearity slope and **(C)** threshold for 200 Monte-Carlo simulations, bracketed by 2× standard deviation, SNR = 10 dB. Parameters of static nonlinearity were estimated by fitting a half-wave rectifier to nonlinearity at each SV.

Figure 7A shows the simulated values of intrinsic pathway elasticity (*K*) as a function of SV in blue and the mean of 200 Monte-Carlo identification estimates bracketed by two standard deviations of the estimates in red. It is evident that mean of estimates were very close to true value with small variance.

Figures 7B,C show the simulated (blue) and estimated (red) slope and threshold of the estimated nonlinearity extracted from 3D nonlinearity. These values were obtained by finding the best half-wave rectifier (HWR) fit to estimated nonlinearity at each SV using Levenberg-Marquardt method in MATLAB curve fitting toolbox. The red curve shows the mean of 200 Monte-Carlo identification estimates bracketed by two standard deviations of the estimates. The mean of the estimates for slope was very close to simulated values showing that we can accurately retrieve the reflex gain. The estimates of thresholds at some SVs were subject to a maximum of 25% error. There are two explanations for this: (1) the simulated model was different from the identified model, i.e., HWR was simulated and Chebyshev polynomials were used for identification. (2) The distribution of input (velocity for reflex pathway) affects the estimation of threshold. The estimates are expected to be more accurate for an input with rectangular probability distribution. However, these choices were made intentionally in this work to evaluate the performance of the algorithm for a practical case, i.e., true nonlinearity may not be the same as that used for identification for physiological systems, and the actuator dynamics affects the input distribution. Nevertheless, the overall estimated threshold variation trend is very close to the true simulated value. Note that since torque has little power at thresholds, the bias in threshold estimate has little effect on torque prediction.

**Figure 8:**
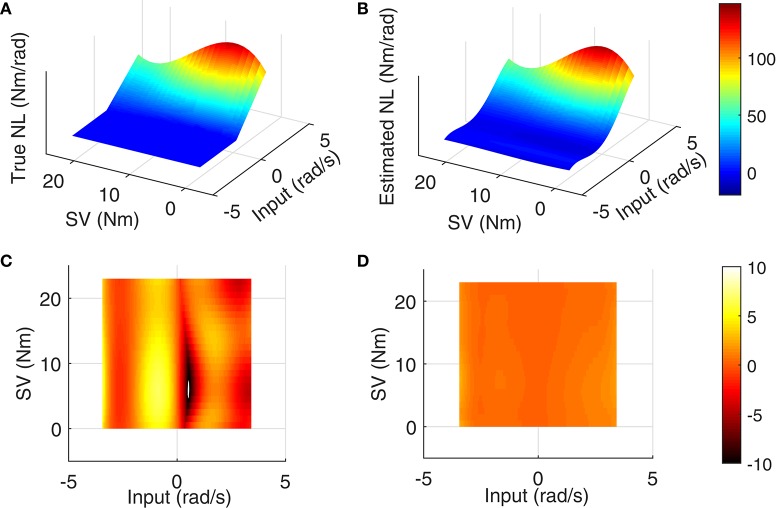
**Reflex static nonlinearity for 200 Monte-Carlo simulation: (A)** true system, **(B)** mean of identification estimates for 200 Monte-Carlo simulations, and top view of 3D plot of **(C)** bias error, and **(D)** random error, SNR = 10 dB.

**Figure 9:**
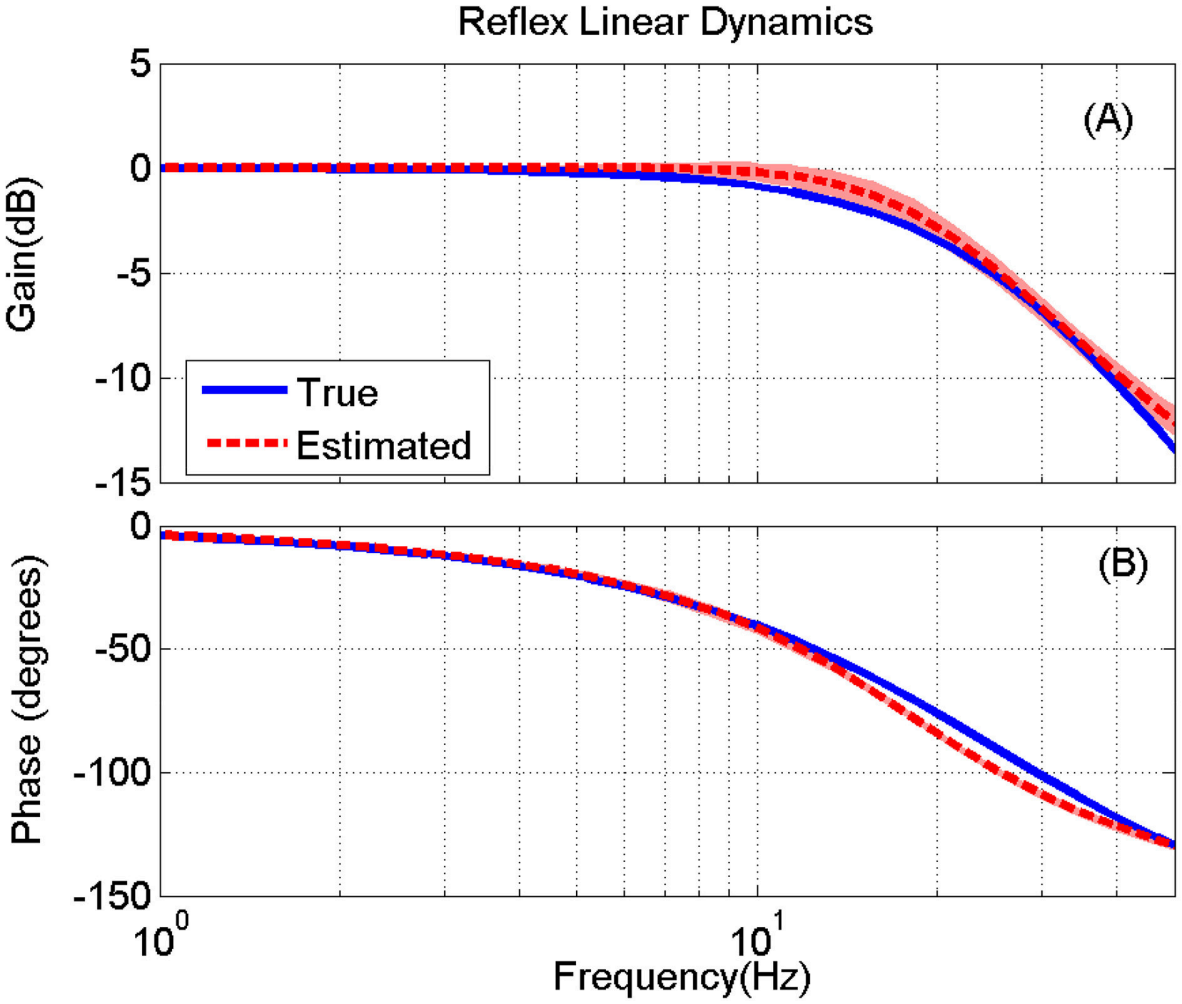
**True (blue) and the mean of estimated (red) reflex linear dynamics (in frequency response representation) (A)** gain and **(B)** phase, for 200 Monte-Carlo simulations, bracketed by 2× standard deviation, SNR = 10 dB. The bode plot is presented up to 50 Hz where the input has enough power for identifications.

The LPV nonlinear block of reflex pathway is plotted in Figure 8 in 3D representation; Figure 8A shows the true simulated nonlinearity whereas the average of 200 estimated nonlinear block is plotted in Figure 8B of this figure. The lower two panels show the bias and random errors for static nonlinearity estimate for 200 simulation trials from top view; both errors were small with maximum bias error occurring around nonlinearity threshold. This is consistent with our estimation of threshold demonstrated in Figure 7C. The maximum bias error was around 10 Nm/rad and the maximum random error was 1 Nm/rad, while the nonlinearity has a maximum gain of 160 Nm/rad. This confirms the efficiency of the proposed algorithm for estimating the LPV static non-linearity. The frequency response of reflex linear dynamic estimate is demonstrated in Figure 9. The linear system was calculated as a subspace system; the frequency response representation is used for better visualization of accuracy at different frequencies. Both the gain and phase estimates were close to true simulated values.

## 4. Experimental study

### 4.1. Methods

The new algorithm was used to characterize the modulation of joint stiffness with activation level in healthy humans performing an isometric torque tracking task of the ankle plantarflexors.

#### 4.1.1. Apparatus

**Figure 10:**
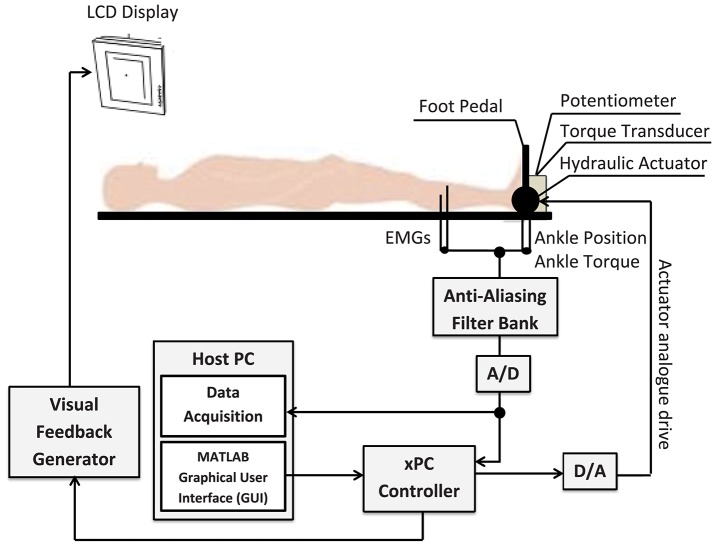
**Schematic of the experimental setup**. The subject's left foot was attached to the actuator pedal by a custom boot. Ankle torque and a target signal were displayed on an overhead monitor. The subject generated dynamic, isometric contractions by tracking the target signal.

Figure 10 shows a schematic of the experimental setup which is described in details in Morier et al. (1990). Subjects lay supine on an experimental table with the left foot attached to a hydraulic actuator using a costume-made fiberglass boot. The neutral position was defined as a 90 degree angle between the foot and shank. Dorsiflexing rotations were taken as positive. The mean ankle angle was set to 0.2 rad.

#### 4.1.2. Subjects

Five healthy subject (one female and four males) aged 26–33 with no history of neuromuscular disorders participated. Subjects gave informed consent to the experimental procedures, which had been reviewed and approved by McGill University Research Ethics Board. Table 1 summarizes the subjects' demographics.

**Table 1:** **Subject characteristics: gender, age, *Maximum Voluntary Contraction* (MVC) torque in *Plantarflexion* (PF), and the normalization factors**.

**Subject**	**Gender**	**Age (years)**	**PF MVC (Nm)**	**Intrinsic elasticity normalization factor**	**Reflex gain normalization factor**	**Reflex delay (ms)**
S1	F	33	26.40	18.24	9.9	45
S2	M	32	55.02	174.06	23.1	45
S3	M	32	43.12	90.94	64	40
S4	M	26	79.25	58.61	95	45
S5	M	33	60.14	126.28	80	40

#### 4.1.3. Data acquisition

EMG signals from tibialis anterior (TA) and triceps surae (TS) including lateral and medial Gastrocnemius muscles were recorded separately using differential surface electrodes. EMGs were amplified and band-pass filtered with a gain of 1,000 and cutoff frequencies 20–2,000 Hz. Ankle torque was low-pass filtered with an eighth-order Bessel filter with cut-off frequency equal to 0.7 Hz in real time and provided to the subject as visual feedback signal. Position, torque and EMG signals were filtered with an anti-aliasing filter at 486.3 Hz, sampled at 1 kHz, and recorded.

#### 4.1.4. Trials

Subjects were instructed to modulate their ankle torque by tracking a visual command signal. The command signal comprised of a sine-wave with a period of 60 s and peak-to-peak amplitude equal to 40% of their maximum voluntary contraction (MVC). Two conditions were examined:

1. *Unperturbed trial* (UT): a low-amplitude pseudo random binary sequence (PRBS) signal was added to the command signal. No position perturbations were applied. The PRBS perturbation was added to command signal (sine-wave) to provide the rich, persistently excitatory input needed for accurate identification of the EMG-Torque dynamics.
2. *Perturbed trial* (PT): random perturbations of ankle position were applied by the hydraulic actuator. The perturbation signal was a PRALDS signal with switching rate of 250–350 ms with amplitude of 0.05 rad.

Data were recorded for 120 s at sampling frequency of 1kHz and then decimated to 100 Hz for analysis. Data were examined for evidence of fatigue or co-activation; there was no evidence of either in any of the trial.

#### 4.1.5. Analysis

Identification was performed in three steps:

1. *EMG-Torque Dynamics Estimation:* We used a time-invariant error-in-variable (EIV) subspace Hammerstein identification algorithm to estimate the dynamic relationship between rectified voluntary Soleus EMG, and torque from UT data. This algorithm provides unbiased estimates of EMG-Torque dynamics in experimental conditions where the feedback is significant as discussed in Golkar and Kearney (2015). This method uses past inputs and outputs as instrumental variables in a manner similar to the subspace Hammerstein identification approach described by Jalaleddini and Kearney (2013).
2. *Estimate of Voluntary Torque in PT trials:* The voluntary component of the EMG was estimated from the EMG record by removing spikes associated with reflex activation. These reflex spikes are generated in response to positive perturbations (muscle stretch). The spikes were located by calculating the derivative of the input perturbation signal (i.e., perturbation velocity) and finding the times where the velocity was large enough to generate a reflex EMG response. The reflex EMG was then replaced by values that linearly interpolated the EMG values preceding and following the spike onset. The voluntary EMG was adopted to the EMG-Torque model identified in step 1 to estimate the voluntary torque (${\hat{TQ}}_{v}$).
3. *Joint Stiffness Identification:* The subspace LPV-PC identification algorithm was used to estimate the Parallel-Cascade system relating ankle position (θ) to the estimated stiffness torque response (${\hat{TQ}}_{s}$) from PT data. The voluntary torque estimated in step 2 was used as the scheduling variable (μ). Stiffness torque (${\hat{TQ}}_{s}$) was estimated by removing the estimated voluntary torque (${\hat{TQ}}_{v}$) from total measured torque (*TQ*_*tot*_).

### 4.2. Results

#### 4.2.1. EMG-torque dynamics estimation

**Figure 11:**
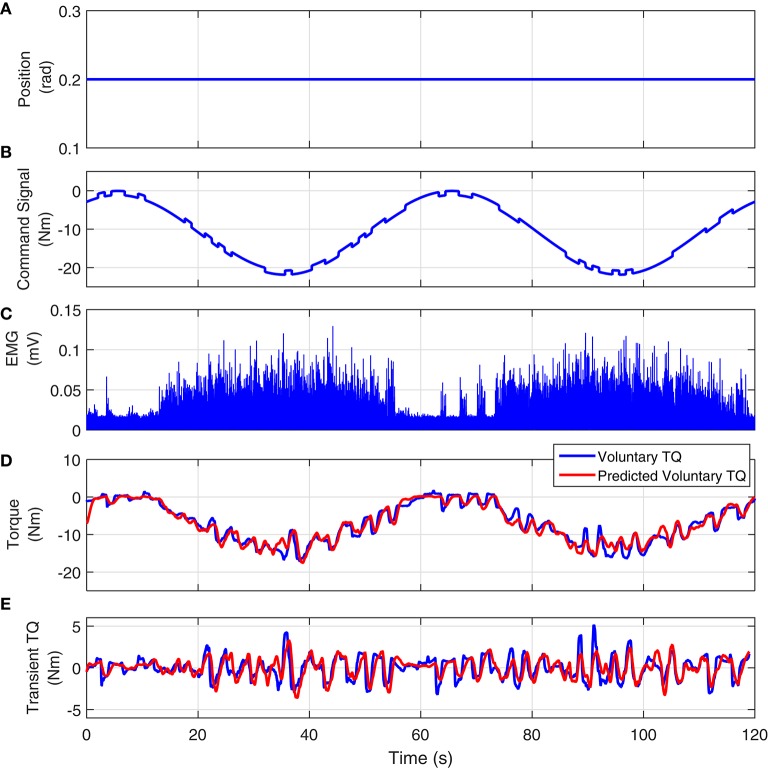
**Typical UT experimental trial from an isometric contraction experiment, Subject S1: (A)** position perturbations, **(B)** visual command signal, **(C)** soleus EMG, **(D)** measured (blue) and predicted (red) ankle torque. The TIV Hammerstein model, estimated between rectified EMG and torque, accurately predicted the voluntary torque with a VAF equal to 93%, **(E)** the transient torque prediction after removing the large slow-varying torque from both measured and predicted torques. The VAF for transient response was 81%.

Figure 11 shows the joint position, visual command, full-wave rectified Soleus EMG and measured and predicted voluntary torque from a typical UT trial. The model estimated between Soleus EMG and torque, predicted the torque extremely well; the variance accounted for was 93% for this subject and 92 ± 3% for all subjects. Figure 11E shows the measured and estimated transient torques. These were obtained by filtering the torques with a moving average Butterworth low-pass filter to remove the slow time-varying torques (sine-wave). The VAF for transient response was 81% for this subject.

#### 4.2.2. Joint stiffness

**Figure 12:**
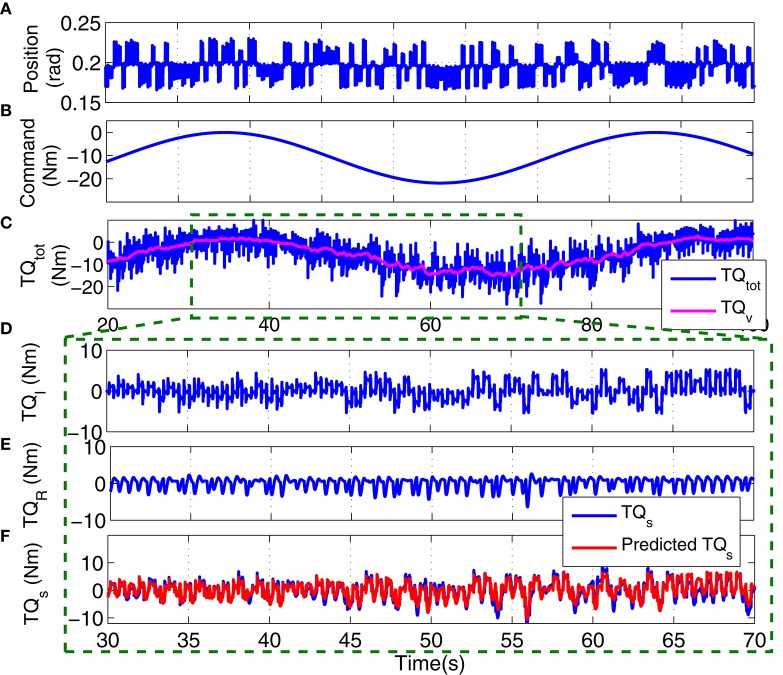
**Typical PT experimental trial from an isometric contraction experiment, Subject S1: (A)** position perturbations, **(B)** visual command signal, **(C)** total torque (blue) and estimated voluntary torque, used as SV of LPV-PC method (magenta), **(D)** identified intrinsic torque, **(E)** identified reflex torque, and **(F)** estimated stiffness torque ($TQ_{tot}-\hat{TQ_{v}}$) (blue) and identified stiffness torque (red). LPV method captured the TV behavior of the system well with a Stiffness VAF of 82% and total VAF (stiffness + voluntary torque) of 95%.

**Figure 13:**
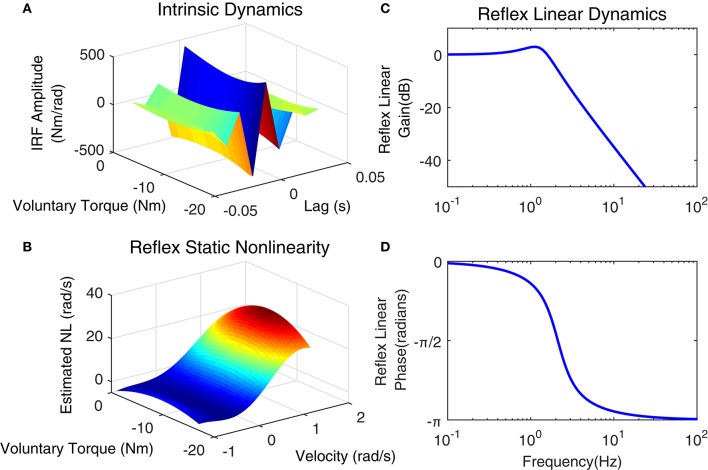
**Identification results of joint stiffness components for a typical subject, Subject 1: (A)** LPV IRF estimated for intrinsic pathway, Reflex pathway: **(B)** estimated nonlinear block as a function of ankle velocity and voluntary torque, Reflex Linear Dynamics frequency representation **(C)** gain, **(D)** phase.

Figure 12 shows the position perturbation, the visual command, and the resulting torque from a typical PT trial. The voluntary torque, estimated from the UT EMG-Torque model is shown in magenta in Figure 12C, superimposed on the total measure torque in blue. The three lower panels show the intrinsic, reflex, and stiffness torques estimated using LPV-PC identification algorithm for the trial segment with largest variation in voluntary torque (SV). Comparing the stiffness torque and that predicted using LPV identification algorithm, it is evident that the LPV method captured the TV behavior of the system well with a VAF of 82% for stiffness torque and 95% for total torque (stiffness + voluntary torque). The total VAF was never <90% in any trial. Figure 13 shows the LPV-PC model estimate for a typical subject. Figure 13A shows the TV behavior of the intrinsic dynamics and how it varies with voluntary torque. Figure 13B shows that the static nonlinearity has a strong uni-directional sensitivity to velocity; the slope varies with voluntary activation increasing from rest to 5 Nm and then decreased. Figures 13C,D show the bode diagram of estimated TIV reflex linear dynamics resembling a second-order low-pass filter system.

**Figure 14:**
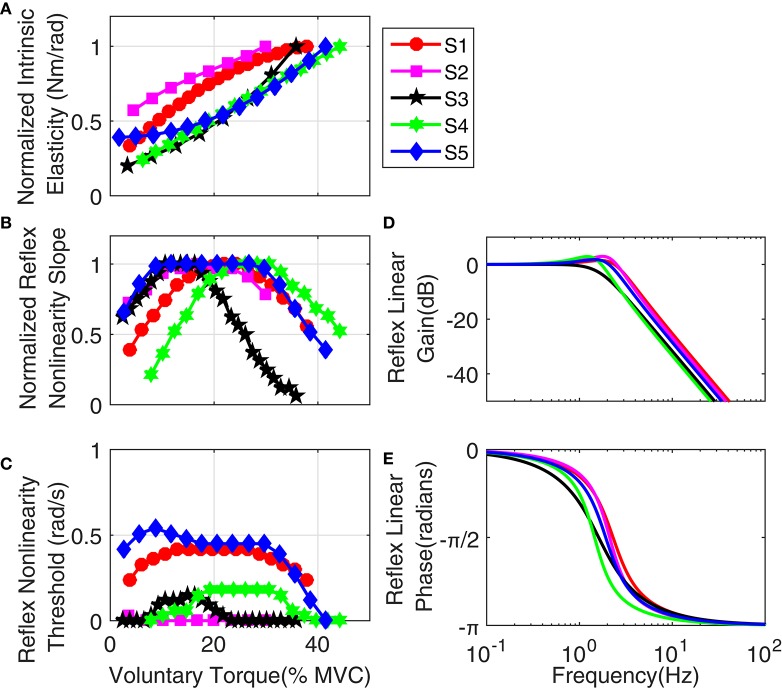
**Group results: (A)** normalized intrinsic elasticity (*K*); this was obtained from the identified LPV IRF of intrinsic stiffness by calculating the steady state value of the integral of identified IRFs, Reflex static nonlinearity: **(B)** normalized gain and **(C)** threshold both changed systematically with activation level. Frequency representation of Reflex Linear Dynamics **(D)** gain, **(E)** phase; reflex linear dynamics was a second-order low-pass filter and cutoff frequency between 1.65 and 2.9 Hz for different subjects.

Figure 14 shows the variation of estimated parameters with voluntary torque for the five subjects. The estimates of intrinsic elasticity and reflex gain (nonlinearity slope) were normalized to their maximum value for the contraction range studied for each subject to allow inter-subject comparison. The original values corresponding to data points in the Figure, can be calculated by multiplying the *x*-axis value by subject's MVC and *y*-axis value by their corresponding normalization factor. The MVC and normalization factors for each subject are given in Table 1. The intrinsic elasticity (*K*) (Figure 14A), monotonically increased with contraction level in all subjects. The reflex gain (Figure 14B) and threshold (Figure 14C) of the static non-linearity systematically changed with voluntary contraction. The reflex gain increased with voluntary torque up to 10–30% MVC in different subjects and then decreased. The variation in reflex gain was higher than 50%. The reflex nonlinearity threshold also varied with voluntary torque and was not always zero as assumed in most quasi-stationary studies. Given the results of the simulation study, the estimates of threshold values may be biased but the overall trends are expected to be informative. The reflex linear block was estimated to be a second-order low-pass filter with delay varying between 40 and 45 ms (see Table 1). Figures 14D,E show the gain and phase of reflex linear dynamics represented in frequency domain. The bandwidth of reflex pathway varies between 1.65 and 2.9 Hz in subjects examined in this work.

## 5. Discussion

This paper investigated and quantified the effects of voluntary contractions on ankle joint dynamic stiffness and its intrinsic and reflex components. Previous work has demonstrated that voluntary muscle activation causes substantial changes of stiffness during functional tasks (Ludvig and Perreault, 2014). Thus, studying this system during large, *continuous* variations in voluntary contraction will lead to better understanding of the control of movement. We used a subspace LPV-PC identification algorithm to track stiffness changes during large, isometric voluntary torque contractions. We first validated the method using a Monte-Carlo simulation study. These demonstrated that the method yielded estimates that were accurate, precise (thus reliable) and capable of capturing time-varying stiffness changes similar to those expected from quasi-stationary results, efficiently. We then applied the method to experimental data acquired while healthy human subjects made large, transient voluntary contractions. Our analysis of these data showed that the stiffness dynamics varied significantly with the contraction. We believe that the system identification algorithm used in this study provides an accurate description of intrinsic and reflex stiffness dynamics throughout a voluntary contraction and so can be used to asses the contribution of each pathway to joint mechanics in functional tasks.

### 5.1. Simulation study

We used simulations of a realistic stiffness model to validate the performance of the subspace LPV-PC identification algorithm when torque varied sinusoidally. The variation in stiffness parameters with torque was obtained by interpolating the results of quasi-stationary experiments with normal human subjects. We used colored output noise with its amplitude adjusted to give an average SNR of 10 dB for each simulation trial. The true experimental noise is expected to be lower than this value (Ludvig et al., 2011). Thus, we evaluated the identification algorithm under condition that is more challenging than that actually seen experimentally. There are two main differences between our simulation study and the experimental conditions: (i) *SV estimation:* In the simulations we assumed that the voluntary torque could be measured and completely removed from total torque. However, in the experiments, the SV must be estimated from the recoded EMG signal. Any errors in estimating the SV will result in identification performance to be lower than that predicted from the simulations. (ii) *Identification model structure:* We made two assumptions about the model structure: (1) Stiffness dynamics at the ankle can be represented using a *PC model structure*; this model has been widely used and shown to be successful in predicting the stiffness torque for both quasi-stationary and TV conditions (Mirbagheri et al., 2000; Sobhani Tehrani et al., 2014; Jalaleddini et al., 2017), (2) The reflex pathway has a *delay* of 40–45ms; this is shown to be true in many studies (Stein and Kearney, 1995; Kearney et al., 1997; Mirbagheri et al., 2000). There were few assumptions about structures of the elements of the PC model. Thus, for the intrinsic pathway the linear dynamics were modeled as a nonparametric IRF whose length was limited to be less than the reflex delay. For the reflex pathway, the nonlinearity is modeled with an orthonormal expansion whose order minimize the prediction error; the linear dynamics were modeled with a parametric model whose order is determined as part of the identification. The excellent prediction ability of the resulting model demonstrates that it accurately reproduces the observed behavior. It is possible that the true structure is more complex than the PC model (i.e., involve more pathways or have complex pathways such as nonlinear-linear-nonlinear cascade). If so, the model is still useful as an approximation since an arbitrary nonlinear system can be represented by a parallel cascade of block structured elements. However, in such a case, there would no longer be a direct relation between the structure of the model and that of the underlying physiological system; this possibility must be taken into account in the interpretation of the results.

### 5.2. Dynamic stiffness

Our experimental results showed that stiffness increased with contraction level suggesting that system became more stiff at high contraction levels. The increase in stiffness may be justified by increase in the number of cross-bridges occurring at higher contraction levels. Reflex gain increased going from rest to lowest active level (occurring between 10 and 20% MVC) and then started to decrease. The variation in reflex gain can be explained by recruitment of more muscle fibers at higher contraction levels and existence of an upper-limit in motoneuron pool excitation. The changes in the nonlinearity threshold suggest changes in motoneuron pool excitation threshold with torque levels. These results indicate that contribution of reflex stiffness is highest at low contractions and decreases as contraction level increase, whereas, intrinsic stiffness monotonically increases with contraction level. Note that we did not attempt to parameterize the LPV IRFs for the intrinsic pathway as a second-order system because: (1) intrinsic dynamics may be more complex than the I,B,K model as demonstrated recently in Sobhani et al. (2017) (2) the fitting procedure would involve non-linear minimization that would introduce an additional source of error.

These findings are essential in understanding the role of stretch reflexes during a motor task particularly those involving low contraction levels such as the control of posture and balance. Other works suggested that intrinsic stiffness is not sufficient to maintain stable upright posture (Morasso and Sanguineti, 2002; Moorhouse and Granata, 2007). Our results show that the range of activation where reflex stiffness is significant, varies among subjects and the reflex contribution was substantial in all subjects examined in this study. Comparing our results to those reported in quasi-stationary condition, the reflex maximum contribution was found to occur around 10% MVC and above whereas this was reported to occur at 5% MVC (Mirbagheri et al., 2000). However, it is not clear whether this is due to the dynamics changes due to task or simply because of differences between the subjects who participated in these studies.

In a separate work, we used a similar approach as that used here to estimate the Hammerstein system of reflex pathway, and evaluated the variation in position-reflex EMG dynamics with contraction levels, in isometric condition (Golkar et al., 2015). It was demonstrated that both gain and threshold of static nonlinearity changed with contraction levels. The results presented in this work combined with that study gives us a comprehensive understanding of how stiffness modulates during isometric TV contractions in plantarflexors of healthy human subjects.

Given the limited dataset required for the subspace LPV-PC identification algorithm used in this study, this can be used toward exploring the effect of some other factors such as contraction history, contraction rate, and contraction trajectory on dynamics of joint stiffness. This can be achieved by repeating the experiment when: (i) the TV torque-matching task starts after a constant activation level is maintained for a short period of time, (ii) use torque-tracking trajectory with different bandwidths (e.g., different periods for sine-wave) as command signal, (iii) use different torque trajectories, e.g., *multi-level*, and compare the estimated models for each case.

### 5.3. Comparison to previous works

The overall trends in our findings agree with the results of quasi-stationary studies. For example, we found that the intrinsic elasticity increased with activation level, similar to the results of Mirbagheri et al. (2000). Also, for reflex gain, We observed a behavior similar to that reported in Jalaleddini et al. (2016). Nonetheless, the magnitudes of the changes were different. We observed 50% increase in stiffness whereas Mirbagheri et al. (2000) reported this to be around 90% for the same range of contraction. Our estimates of reflex gain were similar to those of Mirbagheri et al. (2000), except that we observed a persistence of reflex contribution for a wider range of contraction levels (up to 30% for some subjects). Some other quasi-stationary works reported the maximum reflex contribution to occur around 50% MVC in dorsiflexors (Sinkjaer et al., 1988; Cathers et al., 2004). Based on our experience, this level of activation is very likely to cause fatigue which affects the reliability of results from such experiments. Also, the nominal values reported for maximum reflex contribution based on %MVC might vary among different works due to the differences in measuring the MVCs or the muscle studied.

Van Eesbeek et al. (2013) also used the LPV identification to study wrist stiffness in an activation varying task. However, their method was limited to intrinsic estimates and did not decouple the effects of reflex contribution on the total torque variations. Reflex contributions were reported to be minimal in the upper arm (Bennett et al., 1992) but found to be significant in the ankle (Kearney et al., 1997), wrist (Sinkjær and Hayashi, 1989), and knee (Ludvig and Perreault, 2014). Consequently, the results of Van Eesbeek et al. (2013) cannot be directly compared to ours. Also, the range of activation is very different in the wrist compared to the ankle. Nevertheless, they showed that the main variation in intrinsic parameters at human wrist was in the elastic parameter, variations in viscosity were small and the inertia was found invariant. This is consistent with our results.

Other studies have used ensemble-based method to evaluate the effect of activation level on joint stiffness. Visser (2010) studied ankle joint stiffness during a sinusoidal torque matching task, where a monotonic increase in elastic parameter with voluntary torque was observed similar to the observation of this study. The main difference with our results was that Visser (2010) found two peaks in the reflex gain at the lowest and highest activation levels. Also, Ludvig and Perreault (2014) used a similar ensemble-based method to study knee stiffness during rapid activation and reported similar results for the elastic parameter. Nonetheless, using ensemble-based methods for activation-varying experiments have a number of drawbacks. It requires the exact same time-varying behavior to be repeated many times while (i) it is extremely difficult to match muscle activation levels between trials, (ii) the muscle recruitment strategy might change to avoid fatigue, (iii) antagonist muscle(s) might be activated in some trials to assist the tracking task, (iv) occurrence of fatigue is inevitable especially if activation levels above 30% are used in the study, (v) the desired torque trajectory needs to be slow enough so that subject can repeat the same task many times, and (vi) system behavior may change from the first experiment to the last one considering the large number of trials required.

The LPV identification algorithm described here, models the underlying dependency of system parameters on torque mean and thus should predict the response to novel trajectories for similar conditions. This predictive ability is a strong asset for studying physiological systems. The experiments described here were not designed to demonstrate this ability but are an important next step. In addition, it is not yet known how this predictive ability depends on the temporal and amplitude properties of the SV. This is an important topic for future work.

### 5.4. Limitations of the study

In this study, we used the subspace LPV-PC algorithm and identified a nonlinear model of both intrinsic and reflex ankle stiffness during isometric, time-varying contractions. The model accurately predicted non-stationary torques recorded from experiments with five healthy subjects. In the identified subspace LPV-PC model, the time-varying behavior of the joint was related to background voluntary torque, instead of time, defined as the scheduling variable. Consequently, it provided insight into functional relationships underlying biomechanics of the joint. Also, the model is expected to predict joint response to novel time trajectories of isometric muscle contractions. However, this study has some limitations too, including:

- It assumed that the time-varying behavior of the joint is a function of an *a priori* known scheduling variable. This assumption was valid for the slow isometric contraction experiments of this study. However, may not hold for other situations such as muscle fatigue, rapid contractions, or neuromuscular disorders where the SV is not well known. Similarly, it will almost certainly not hold in functional tasks where stiffness parameters depend on multiple variables. For example, during most movements both torque and position change; stiffness parameters are known to depend strongly on both, so it is to be expected that modeling this behavior would require at least two SVs.
- Reflex linear dynamics were assumed to be time-invariant except for its gain that can be modeled by the LPV nonlinearity. This seems to be a valid assumption for healthy subjects performing isometric, slow time-varying contractions (for the contraction range studied in this study) or large imposed movement at rest (Sobhani Tehrani et al., 2014; Jalaleddini et al., 2015). However, it may not be valid for pathological subjects whose reflex dynamics have been shown to change with contraction level (Mirbagheri et al., 2001). Nevertheless, if the subspace LPV-PC identification algorithm is used to analyze a system with TV reflex dynamics, the estimates of intrinsic pathway and corresponding interpretations should remain almost intact. This is because, the subspace LPV-PC identification algorithm uses an orthogonal projection approach to decompose the torque into intrinsic and reflex torques. Thus, any inaccuracy in system structure assumed for reflex dynamics is not expected to affect the estimates of intrinsic dynamics. Rather it would bias estimates of reflex nonlinearity and result in a decrease in torque VAF. Sobhani Tehrani (2017) recently has developed a non-parametric LPV-PC method that can identify SV-dependent changes in reflex dynamics. Future work will use this to investigate the importance of TV changes in reflex dynamics and if this improves the predictions.
- The model parameters are assumed to be static functions of the SV while *dynamic* dependencies may occur in some functional tasks. For the slow isometric contraction trajectory used in this work, the static dependency assumption is expected to be valid. The VAF of its predicted torques supports this assumption. However, assumption must be validated for rapidly changing contractions. In general, if the model parameters depend dynamically on the SV, the LPV identification algorithm would not be expected to predict well. We are not aware of any work investigating potential dynamic dependencies between voluntary torque and joint stiffness parameters. Indeed, the subspace LPV-PC identification algorithm provided the tool needed to investigate such dependencies.
- Since the voluntary torque (i.e., the SV) is not directly measurable, we estimated it using an EMG-Torque Hammerstein model, identified from experimental data. The *risk* is that inaccuracies in the EMG-torque model, and thus the estimated scheduling variable, may bias the identified LPV stiffness model parameters.

Finally, note that this study was performed under open-loop experimental conditions, where the perturbing actuator was many times more stiffness than the ankle. Consequently, the torque generated at the ankle could not change the position of the actuator. This is not the case when subjects interact with compliant loads, where closed-loop conditions may arise. The subspace family of identification algorithms are believed to work with data acquired in closed-loop conditions (Van Wingerden and Verhaegen, 2009); however, validating this with experimental data acquired specifically for LPV-PC modeling of joint stiffness is a subject of future work.

### 5.5. Clinical significance

The subspace LPV-PC method would be an invaluable tool for objective and quantitative assessment of neuromuscular performance (or impairment) and motor function (or dysfunction). In fact, the early signs of recognizing the clinical benefits of exploiting system identification and modeling approach have recently appeared in the literature (Meskers et al., 2015; Sloot, 2016), where, for example, system identification was used to assess motor dysfunction in children with cerebral palsy. The subspace LPV-method can actually enable and expedite this shift from conventional scoring techniques to *model-based* clinical assessment, diagnosis, and treatment recommendation. Few of the reasons are:

- It works for much more functional tasks compared to quasi-stationary studies. In addition, the identified LPV model is not just a predictive model. Rather, it provides a coherent representation of the joint biomechanics where the systematic changes are functionally related to variables within the neuromuscular system.
- It is far more efficient than the quasi-stationary methods because it requires many fewer trials. For example, in the isometric TV contraction experiment of this study, we used only two trials (UT and PT) to identify the LPV-PC model; whereas the quasi-stationary studies require many more trials to cover the same range of activation levels with a fine resolution. For example, 11 trials are needed to cover activation levels from rest to 40% MVC with a resolution of 2% MVC; thus the LPV method reduces the required number of trials by more than 80%. Such reductions are of utmost importance and value working with patients and in clinical applications.
- By estimating the individual elements of the subspace LPV-PC stiffness model, the method distinguishes between the mechanical and reflex contributions to the abnormal joint mechanics, which is very important from a clinical standpoint. Thus, the method will have significant clinical benefits for diagnosis and treatment monitoring of patients suffering from neuromuscular diseases such as cerebral palsy, spinal cord injury, stroke, and Parkinson's disease.

## Ethics statement

This study was carried out in accordance with the recommendations of McGill University Research Ethics Board with written informed consent from all subjects. All subjects gave written informed consent in accordance with the Declaration of Helsinki. The protocol was approved by the McGill University Research Ethics Board.

## Author contributions

MAG implemented the simulation study, collected the experimental data, and performed analysis on experimental data. EST developed the identification algorithm. MAG and EST contributed to the execution and drafting of this paper and the work was supervised, reviewed, and approved by REK.

### Conflict of interest statement

The authors declare that the research was conducted in the absence of any commercial or financial relationships that could be construed as a potential conflict of interest.

## References

[B1] Amato M. P. Ponziani G. (1999). Quantification of impairment in ms: discussion of the scales in use. Mult. Scler. J. 5, 216–219. 10.1177/135245859900500404 10467378

[B2] Bar-On L. Desloovere K. Molenaers G. Harlaar J. Kindt T. Aertbeliën E. (2014). Identification of the neural component of torque during manually-applied spasticity assessments in children with cerebral palsy. Gait & Posture 40, 346–351. 10.1016/j.gaitpost.2014.04.207 24931109

[B3] Bennett D. J. Hollerbach J. Xu Y. Hunter I. (1992). Time-varying stiffness of human elbow joint during cyclic voluntary movement. Exp. Brain Res. 88, 433–442. 10.1007/BF02259118 1577114

[B4] Carter R. R. Crago P. E. Keith M. W. (1990). Stiffness regulation by reflex action in the normal human hand. J. Neurophysiol. 64, 105–118. 2388060 10.1152/jn.1990.64.1.105

[B5] Cathers I. O'Dwyer N. Neilson P. (2004). Variation of magnitude and timing of wrist flexor stretch reflex across the full range of voluntary activation. Exp. Brain Res. 157, 324–335. 10.1007/s00221-004-1848-7 15007580

[B6] Cerone V. Piga D. Regruto D. Berehanu S. (2012). LPV identification of the glucose-insulin dynamics in type i diabetes, in Proceedings of the 16th IFAC Symposium on System Identification (Brussels), 559–564.

[B7] de Vlugt E. de Groot J. H. Schenkeveld K. E. Arendzen J. van der Helm F. C. Meskers C. G. (2010). The relation between neuromechanical parameters and ashworth score in stroke patients. J. Neuroeng. Rehabil. 7:35. 10.1186/1743-0003-7-35 20663189 PMC2927906

[B8] Golkar M. A. Jalaleddini K. Tehrani E. S. Kearney R. E. (2015). Identification of time-varying dynamics of reflex EMG in the ankle plantarflexors during time-varying, isometric contractions, in 37th Annual International Conference of the IEEE Engineering in Medicine and Biology Society (EMBC) (Milan), 6744–6747. 10.1109/EMBC.2015.7319941 26737841

[B9] Golkar M. A. Kearney R. E. (2015). Closed-loop identification of the dynamic relation between surface EMG and torque at the human ankle, in Proceeding of 17th IFAC Symposium on System Identification (Beijing), 263–268.

[B10] Guarin D. L. Kearney R. E. (2015). Time-varying identification of ankle dynamic joint stiffness during movement with constant muscle activation, in 37th Annual International Conference of the IEEE Engineering in Medicine and Biology Society (EMBC) (Milan), 6740–6743. 10.1109/EMBC.2015.7319940 26737840

[B11] Ikharia B. I. Westwick D. T. (2006). Identification of time-varying hammerstein systems using a basis expansion approach, in Canadian Conference on Electrical and Computer Engineering (CCECE06) (Ottawa), 1858–1861.

[B12] Ikharia B. I. Westwick D. T. (2007). On the identification of hammerstein systems with time-varying parameters, in 29th Annual International Conference of the IEEE Engineering in Medicine and Biology Society (EMBC) (Lyon), 6475–6478. 10.1109/IEMBS.2007.4353842 18003508

[B13] Jalaleddini K. Golkar M. A. Guarin D. L. Tehrani E. S. Kearney R. E. (2015). Parametric methods for identification of time-invariant and time-varying joint stiffness models, in Proceeding of 17th IFAC Symposium on System Identification (Beijing), 1375–1380.

[B14] Jalaleddini K. Golkar M. A. Kearney R. E. (2017). Measurement of dynamic joint stiffness from multiple short data segments. IEEE Trans. Neural Syst. Rehabil. Eng. [Epub ahead of print]. 10.1109/TNSRE.2017.2659749 28278472

[B15] Jalaleddini K. Kearney R. E. (2013). Subspace identification of SISO Hammerstein systems: application to stretch reflex identification. IEEE Trans. Biomed. Eng. 60, 2725–2734. 10.1109/TBME.2013.2264216 23708763

[B16] Jalaleddini K. Kearney R. E. (2011). Estimation of the gain and threshold of the stretch reflex with a novel subspace identification algorithm, in 2011 Annual International Conference of the IEEE Engineering in Medicine and Biology Society (EMBC) (Boston, MA), 4431–4434. 10.1109/IEMBS.2011.6091099 22255322

[B17] Jalaleddini K. Tehrani E. S. Kearney R. E. (2016). A subspace approach to the structural decomposition and identification of ankle joint dynamic stiffness. IEEE Trans. Biomed. Eng. [Epub ahead of print]. 10.1109/TBME.2016.2604293 28113221

[B18] Javed F. Savkin A. V. Chan G. S. MacKie J. D. Lovell N. H. (2010). Linear parameter varying system based modeling of hemodynamic response to profiled hemodialysis, in 32nd Annual International Conference of the IEEE Engineering in Medicine and Biology Society (EMBC) (Buenos Aires), 4967–4970. 10.1109/IEMBS.2010.5627221 21096674

[B19] Kearney R. E. Stein R. B. Parameswaran L. (1997). Identification of intrinsic and reflex contributions to human ankle stiffness dynamics. IEEE Trans. Biomed. Eng. 44, 493–504. 10.1109/10.581944 9151483

[B20] Kirsch R. F. Kearney R. E. (1997). Identification of time-varying stiffness dynamics of the human ankle joint during an imposed movement. Exp. Brain Res. 114, 71–85. 10.1007/PL00005625 9125453

[B21] Kirsch R. F. Kearney R. E. MacNeil J. B. (1993). Identification of time-varying dynamics of the human triceps surae stretch reflex. Exp. Brain Res. 97, 115–127. 10.1007/BF00228822 8131823

[B22] Lee H. Hogan N. (2015). Time-varying ankle mechanical impedance during human locomotion. IEEE Trans. Neural Syst. Rehabil. Eng. 23, 755–764. 10.1109/TNSRE.2014.2346927 25137730

[B23] Ludvig D. Perreault E. J. (2012). System identification of physiological systems using short data segments. IEEE Trans. Biomed. Eng. 59, 3541–3549. 10.1109/TBME.2012.2220767 23033429 PMC3601444

[B24] Ludvig D. Perreault E. J. (2014). The dynamic effect of muscle activation on knee stiffness, in 36th Annual International Conference of the IEEE Engineering in Medicine and Biology Society (EMBC) (Chicago), 1599–1602. 10.1109/EMBC.2014.6943910 PMC6219385 25570278

[B25] Ludvig D. Visser T. S. Giesbrecht H. Kearney R. E. (2011). Identification of time-varying intrinsic and reflex joint stiffness. IEEE Trans. Biomed. Eng. 58, 1715–1723. 10.1109/TBME.2011.2113184 21317071

[B26] MacNeil J. B. Kearney R. Hunter I. (1992). Identification of time-varying biological systems from ensemble data (joint dynamics application). IEEE Trans. Biomed. Eng. 39, 1213–1225. 10.1109/10.184697 1487284

[B27] Meskers C. G. de Groot J. H. de Vlugt E. Schouten A. C. (2015). Neurocontrol of movement: system identification approach for clinical benefit. Front. Integr. Neurosci. 9:48. 10.3389/fnint.2015.00048 26441563 PMC4561669

[B28] Mirbagheri M. Barbeau H. Kearney R. (2000). Intrinsic and reflex contributions to human ankle stiffness: variation with activation level and position. Exp. Brain Res. 135, 423–436. 10.1007/s002210000534 11156307

[B29] Mirbagheri M. Barbeau H. Ladouceur M. Kearney R. (2001). Intrinsic and reflex stiffness in normal and spastic, spinal cord injured subjects. Exp. Brain Res. 141, 446–459. 10.1007/s00221-001-0901-z 11810139

[B30] Mohammadpour J. Scherer C. W. (2012). Control of Linear Parameter Varying Systems with Applications. New York, NY: Springer.

[B31] Moorhouse K. M. Granata K. P. (2007). Role of reflex dynamics in spinal stability: intrinsic muscle stiffness alone is insufficient for stability. J. Biomech. 40, 1058–1065. 10.1016/j.jbiomech.2006.04.018 16782106 PMC1851677

[B32] Morasso P. G. Sanguineti V. (2002). Ankle muscle stiffness alone cannot stabilize balance during quiet standing. J. Neurophysiol. 88, 2157–2162. 10.1152/jn.00719.2001 12364538

[B33] Morier R. Weiss P. Kearney R. (1990). Low inertia, rigid limb fixation using glass fibre casting bandage. Med. Biol. Eng. Comput. 28, 96–99. 2325459 10.1007/BF02441686

[B34] Palazzolo J. J. Ferraro M. Krebs H. I. Lynch D. Volpe B. T. Hogan N. (2007). Stochastic estimation of arm mechanical impedance during robotic stroke rehabilitation. IEEE Trans. Neural Syst. Rehabil. Eng. 15, 94–103. 10.1109/TNSRE.2007.891392 17436881 PMC2752649

[B35] Rouse E. J. Hargrove L. J. Perreault E. J. Kuiken T. A. (2014). Estimation of human ankle impedance during the stance phase of walking. IEEE Trans. Neural Syst. Rehabil. Eng. 22, 870–878. 10.1109/TNSRE.2014.2307256 24760937 PMC5823694

[B36] Sanyal S. Kukreja S. L. Perreault E. J. Westwick D. T. (2005). Identification of linear time varying systems using basis pursuit, in 27th Annual International Conference of the IEEE Engineering in Medicine and Biology Society (EMBC) (Shanghai), 22–25. 10.1109/IEMBS.2005.1616332 17282101

[B37] Sartori M. MacUlan M. Pizzolato C. Reggiani M. Farina D. (2015). Modeling and simulating the neuromuscular mechanisms regulating ankle and knee joint stiffness during human locomotion. J. Neurophysiol. 114, 2509–2527. 10.1152/jn.00989.2014 26245321 PMC4620138

[B38] Sinkjaer T. Andersen J. B. Larsen B. (1996). Soleus stretch reflex modulation during gait in humans. J. Neurophysiol. 76, 1112–1120. 8871224 10.1152/jn.1996.76.2.1112

[B39] Sinkjær T. Hayashi R. (1989). Regulation of wrist stiffness by the stretch reflex. J. Biomech. 22, 1133–1140. 10.1016/0021-9290(89)90215-7 2625413

[B40] Sinkjaer T. Toft E. Andreassen S. Hornemann B. C. (1988). Muscle stiffness in human ankle dorsiflexors: intrinsic and reflex components. J. Neurophysiol. 60, 1110–1121. 3171659 10.1152/jn.1988.60.3.1110

[B41] Sloot L. H. (2016). Advanced Technologies to Assess Motor Dysfunction in Children with Cerebral Palsy. Ph.D. Thesis, Vrije Universiteit Amsterdam, Amsterdam.

[B42] Sobhani Tehrani E. (2017). Linear Parameter Varying Identification of Nonlinear Physiological Systems: Application to Ankle Joint Biomechanics. Ph.D. thesis, Department of Biomedical Engineering, McGill University, Montreal, QC.

[B43] Sobhani Tehrani E. Jalaleddini K. Kearney R. E. (2013a). Linear parameter varying identification of ankle joint intrinsic stiffness during imposed walking movements, in 35th Annual International Conference of the IEEE Engineering in Medicine and Biology Society (EMBC) (Osaka) 4923–4927. 10.1109/EMBC.2013.6610652 24110839

[B44] Sobhani Tehrani E. Jalaleddini K. Kearney R. E. (2013b). A novel algorithm for linear parameter varying identification of hammerstein systems with time-varying nonlinearities, in 35th Annual International Conference of the IEEE Engineering in Medicine and Biology Society (EMBC) (Osaka), 4928–4932. 10.1109/EMBC.2013.6610653 24110840

[B45] Sobhani Tehrani E. Jalaleddini K. Kearney R. E. (2014). Identification of ankle joint stiffness during passive movements-a subspace linear parameter varying approach, in 36th Annual International Conference of the IEEE Engineering in Medicine and Biology Society (EMBC) (Chicago) 1603–1606. 10.1109/EMBC.2014.6943911 25570279

[B46] Sobhani Tehrani E. Jalaleddini K. Kearney R. E. (2017). Ankle joint intrinsic dynamics is more complex than a mass-spring-damper model. IEEE Trans. Neural Syst. Rehabil. Eng. [Epub ahead of print]. 10.1109/TNSRE.2017.2679722 28287979

[B47] Stein R. Kearney R. (1995). Nonlinear behavior of muscle reflexes at the human ankle joint. J. Neurophysiol. 73, 65–72. 7714590 10.1152/jn.1995.73.1.65

[B48] Van der Helm F. C. T. Schouten A. C. de Vlugt E. Brouwn G. G. (2002). Identification of intrinsic and reflexive components of human arm dynamics during postural control. J. Neurosci. Methods 119, 1–14. 10.1016/S0165-0270(02)00147-4 12234629

[B49] Van Eesbeek S. van der Helm F. Verhaegen M. de Vlugt E. (2013). LPV subspace identification of time-variant joint impedance, in Proceedings of the 6th International IEEE/EMBS Conference on Neural Engineering (NER) (San Diego, CA), 343–346.

[B50] Van Wingerden J.-W. Verhaegen M. (2009). Subspace identification of bilinear and lpv systems for open-and closed-loop data. Automatica 45, 372–381. 10.1016/j.automatica.2008.08.015

[B51] Verhaegen M. Dewilde P. (1992). Subspace model identification part 1. the output-error state-space model identification class of algorithms. Int. J. Control 56, 1187–1210. 10.1080/00207179208934363

[B52] Visser S. (2010). Evaluation and Application of an Algorithm for the Time-Varying Identification of Ankle Stiffness. Masters Thesis, Department of Biomedical Engineering, McGill University, Montreal, QC.

[B53] Weiss P. L. Kearney R. Hunter I. (1986). Position dependence of ankle joint dynamics -II. active mechanics. J. Biomech. 19, 737–751. 3793748 10.1016/0021-9290(86)90197-1

